# Nanomagnetic Elastomers for Realizing Highly Responsive Micro- and Nanosystems

**DOI:** 10.1021/acs.nanolett.3c00819

**Published:** 2023-07-19

**Authors:** Bhavana B. Venkataramanachar, Jianing Li, Tanveer ul Islam, Ye Wang, Jaap M. J. den Toonder

**Affiliations:** † Microsystems Section, Mechanical Engineering, Eindhoven University of Technology, Eindhoven 5612 AZ, The Netherlands; ‡ Institute for Complex Molecular Systems, Eindhoven University of Technology, Eindhoven 5612 AZ, The Netherlands; § Department of Applied Physics and Science Education, Eindhoven University of Technology, Eindhoven 5612 AZ, The Netherlands

**Keywords:** magnetic elastomers, responsive nanostructures, highly compliant polymers, artificial cilia

## Abstract

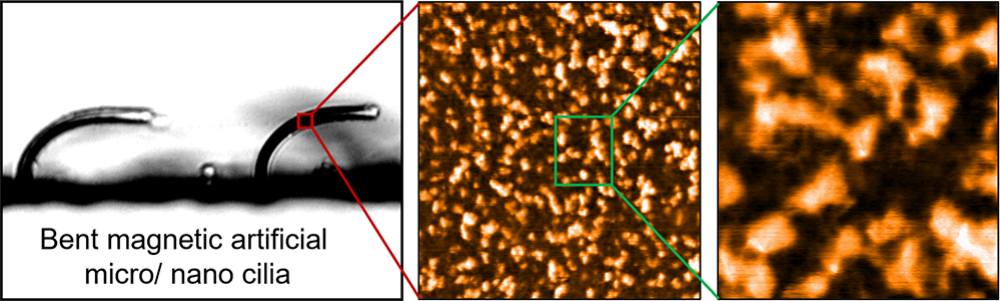

Evolution has produced natural systems that generate motion and sense external stimuli at the micro- and nanoscales. At extremely small scales, the intricate motions and large deformations shown by these biosystems are due to a tipping balance between their structural compliance and the actuating force generated in them. Artificially mimicking such ingenious systems for scientific and engineering applications has been approached through the development and use of different smart materials mostly limited to microscale dimensions. To push the application range down to the nanoscale, we developed a material preparation process that yields a library of nanomagnetic elastomers with high magnetic particle concentrations. Through this process, we have realized a material with the highest magnetic-to-elastic force ratio, as is shown by an extensive mechanical and magnetic characterization of the materials. Furthermore, we have fabricated and actuated micro- and nanostructures mimicking cilia, demonstrating the extreme compliance and responsiveness of the developed materials.

The bioworld is replete with a vast range of micro- and nanoscale systems functioning as actuators and sensors. Examples of bioactuators at the cellular scale are myocytes in the heart and muscle tissues, which induce actuation at the cellular/micro level first and then at an organ level when working as a group. At nanoscales, the hair like organelle called cilium, which exists ubiquitously on the surface of cells in our body, is an example of a fundamental bioactuator and a versatile biosensor. With a minimum length of just a few micrometers and a diameter of a few hundred nanometers,^1^ the cilia show extreme deflection while acting as an actuator and great compliance while functioning as a sensor.^2^ The pursuit of mimicking such systems over the last two decades^3^ has led to numerous inventions and to a deeper understanding of how various biosystems work.^4^ As an example, the biomimicry of cilia has found applications in areas extending beyond their inherent function, like fluid mixing, generating fluid flow, manipulating particles, and sensing fluid flow in microfluidic platforms like lab-on-a-chip and organ-on-a-chip devices.^5^

A range of responsive materials have been developed and employed either for biomimicking or for micro- and nanoengineering applications, such as light sensitive materials,^6,7^ pH based gels and polymers,^8^ electrically actuated MEMS materials,^9,10^ and the most prominent magnetic materials.^11−14^ Greater acceptance of magnetic materials over all other materials is due to the combined properties of instantaneous response to external stimuli, remote access and actuation, biocompatibility, and high elasticity that these materials offer.^15,16^ Exploiting their highly elastic nature, mimicking compliant biosystems at submicrometer scales like cilia has only begun to be realized in the past few years.^14^ Beyond biomimicry, the development of magnetic materials which can allow control and shape transitions like bending and twisting at submicrometer scales is of utmost importance in the field of soft micro- and nanorobots, drug delivery, cell manipulation, minimally invasive surgery, and more.^17−19^

Magnetic materials, also referred to as magneto-rheological (MR) materials, are smart materials often containing magnetic constituents dispersed in a nonmagnetic medium and are further distinguished as MR gels, MR foams, MR fluids, and MR elastomers or magnetic elastomers.^20^ Ever since the beginning of the development of magnetic elastomers,^21,22^ the most commonly used types of magnetic particles have had a size in the micrometer range and are found to exhibit enhanced magnetic properties.^23^ A nonmagnetic polymer like polydimethylsiloxane (PDMS) or epoxy^23,24^ forms the elastic base in which these magnetic particles are embedded through various methods and in different forms, giving a responsive property to the elastic material.^25^ The micrometer scale of the magnetic particles in the elastomer renders them incompatible with realizing submicrometer structures, which would require magnetic nanoparticles. However, a major limitation of magnetic nanoparticles is their much lower magnetizing potential compared to the bigger size microparticles.^23,26^ To account for the lower magnetization, increasing particle concentration through various methods has either resulted in the agglomeration of the particles^27,28^ which hampers their use in micro- and nanoapplications, or in a higher modulus of elasticity limiting the material’s responsiveness.^13^ Since the addition of particles increases the modulus, the maximum particle concentration is restricted below a small value of 18% by weight.^28,29^ Embedding particles in a polymer with typically shorter molecular chains, to increase the particle concentration while preventing agglomeration, has required the use of chemical processes that again impart a high rigidity to the material.^13^ The ratio of the degree of material magnetization to the elastic modulus, referred to as the magnetoelastic ratio (MER), represents the extent to which a material can deform under the application of an external magnetic field.^30,31^ In order to achieve extreme deformation for attaining a higher degree of mobility and enabling complex task performing abilities to a designed micro- or nanosystem, a material with a high magnetoelastic ratio is favored. A higher MER is further central to developing effective small scale sensors like flow sensors in a microfluidic channel.^11,32,33^

Appreciating the importance of polymer molecular chain lengths in preparing responsive nanomagnetic elastomers and therefore employing polymers of shorter molecular lengths, we report here the synthesis of a range of nanomagnetic elastomers with the highest magnetoelastic ratios suitable for realizing highly responsive and compliant micro- and nanosystems. We further report an extensive and detailed mechanical and magnetic property characterization showing a clear trend with magnetic content and with variation of the base molecular chain lengths. To demonstrate the compliance and responsiveness of the materials, simple cylindrical micro- and nanohairs, mimicking cilia, were fabricated and actuated by applying a magnetic torque upon which the cilia showed a maximum deformation. These materials extend the range of control and responsiveness of smart materials at submicrometer scales, and we envision that they will open up new applications for micro- and nanoactuation and sensing.

The polymers chosen as the base materials for the synthesis of the nanomagnetic elastomers have a very low molecular weight (*M*_w_) of 2000–9000 g/mol. This is a representative of shorter molecular chain lengths as compared to the traditionally used polymers, like PDMS, which can have 100′*s* of times higher molecular weights.^34^ Also, the polymer molecules contain an amine group as aminopropylmethylsiloxane (AMS) attached to a chain of dimethylsiloxane (DMS) that forms the backbone of the polymer molecules. The amine acts as a functional group that binds with a magnetic nanoparticle under specific conditions to form a layer/coating around the particle. Coating of the particles is essential in avoiding their agglomeration and achieving a uniform distribution in the base polymer at the required nanoscales. Different concentration ranges of AMS present in DMS of three different forms, obtained from Gelest Inc., make polymers with average molecular weights of 2500, 4500, and 8000 g/mol and are commercially referred to as AMS-191, AMS-162, and AMS-152 respectively. The three polymers with the given molecular weights are apt for preparing nanomagnetic elastomers covering a large range of magnetoelastic properties, see Table 1.

**Table 1:** Elastic Moduli and Time Constants from the Viscoelastic Model Fit, Eq 1, to the Data for Our Nanomagnetic Elastomers, For Three Different Base Polymers and Four Different Magnetic Nanoparticle Concentrations

	FF-AMS-191 MW = 2500 g/mol			FF-AMS-162 MW = 4500 g/mol			FF-AMS-152 MW = 8000 g/mol		
Magnetic content (wt %)	*E*_1_ (MPa)	*E*_2_ (MPa)	τ (s)	*E*_1_ (MPa)	*E*_2_ (MPa)	τ (s)	*E*_1_ (MPa)	*E*_2_ (MPa)	τ (s)
10	0.07	0.12	81	0.02	0.23	78	0.42	0.15	142
20	0.11	0.16	81	0.08	0.38	95	0.58	0.17	145
30	0.33	0.41	86	0.36	0.58	81	1.06	0.25	150
40	0.77	0.46	109	0.45	0.86	85	1.52	0.22	150

The magnetic nanoparticles to be embedded with the polymers were first synthesized by titrating an acidic solution (pH ∼ 2) of ferric and ferrous chloride with an ammonium hydroxide solution to precipitate the nanoparticles of ferric oxide*-*magnetite particles,^28,35,36^ see Figure 1a. After precipitation of the magnetic nanoparticles, continued addition of ammonium hydroxide was used to increase the solution pH to a maximum value of around 12, see Figure 1b. A slow increase in pH with respect to the addition of ammonium hydroxide was used to precisely control the solution pH at different critical values between 9–12. Right pH conditions were needed to facilitate electrostatic binding/coating^13,37^ between positively charged magnetic nanoparticles and the negatively charged amine group of the polymer molecules. Each of the three polymers was found to show maximum binding with the particles above a certain critical pH value, which scaled inversely with the polymer molecular weight; see Figure 1b. Below the critical pH values, a much reduced amount of magnetic nanoparticles got coated, see Figure 1b, whereas above the critical values, a very small percentage of the particles were left uncoated and were removed by a subsequent sedimentation process, see Figure 1a. Since for a relatively low molecular weight polymer a high pH value was needed, and vice versa, which was achieved by adding more ammonium hydroxide solution, an increased quantity of the binding polymer was also added to maintain an optimum concentration in the solution, see Text S1. A higher concentration of a polymer in the solution containing magnetic nanoparticles increased the interacting events between them during a long 14–16 h of stirring process in an inert environment to achieve maximum particle–polymer binding. The coated particles were separated from the solution and their concentration was precisely adjusted at any value below 40 wt %. The maximum value of 40 wt % is determined by the percentage of the material that forms the coated layer on a particle which is equivalent to 50–60 wt %, depending on the molecular weight of the polymer, and is therefore the maximum achievable concentration. A step-by-step process of synthesizing the magnetic nanoparticles, their extraction from the solutions and separation from the uncoated ones, adjusting their concentration in the base polymers to first get ferrofluids and then their curing to get the final magnetic elastomers, is described in detail in the Text S1 and Figure S1 and S2. The homogeneity of the distribution of the nanoparticles in the polymer matrix is shown by scanning electron microscopy (SEM) and atomic force microscopy (AFM) images in Figure S3a, b; the particle size is approximately 20 nm.

**Figure 1:**
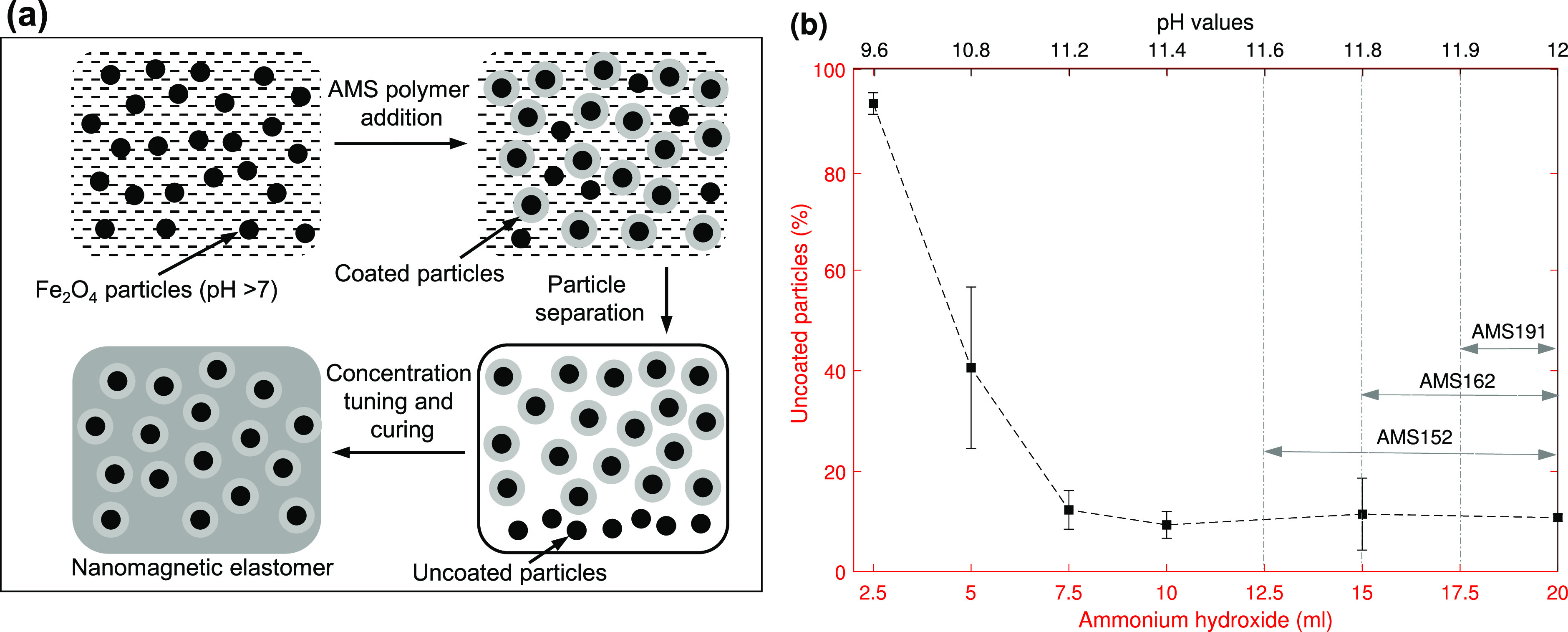
Material synthesis: (a) Schematically represented; first magnetite nanoparticles are precipitated by titrating iron salts, and then a polymer (aminopropylmethylsiloxane, AMS) is added to coat the magnetic nanoparticles during a long stirring process. The particles are then separated from the solution through an intensive washing process and a small percentage of magnetic nanoparticles left uncoated sediments. Finally, the concentration of magnetic particles is tuned by adding a calculated amount of base polymer which is then cured to get the nanomagnetic elastomer. Details of the material preparation process are given in Text S1 and Figures S1 and S2. (b) Increase/decrease in particle coating/uncoating with the increase/decrease in pH value of the solution containing magnetic nanoparticles is first characterized using the polymer AMS-162 and the most favorable pH condition of 11.8–12 is found (top *x*-axis). For the other two polymers with minimum (AMS-191) and maximum (AMS-152) molecular weight among the polymers used in this study, the most favorable conditions exist at pH values of 11.9–12 and 11.6–12, respectively. Although the variation in pH is very small, between 11.6 and 12, this change happens by adding a large quantity of ammonium hydroxide solution (7.5 mL), which helps in maintaining a precise pH condition (bottom *x*-axis).

Toward testing the elastic properties of the elastomers, the ferrofluids (FF) yielded by the material synthesis process were used as precursors and were tuned to different concentrations of magnetic particles by wt %. The ferrofluids were cured in bulk volumes in a vacuum oven at 130 °C for 8–9 h; the obtained materials with different molecular weights are referred to as FF-AMS-191, FF-AMS-162, and FF-AMS-152. From the bulk volume, test samples shaped as rectangular blocks were cut out using a flat edged blade mounted on an XYZ platform to get defect-free and right-angled sides and edges of the sample blocks; see Figure 2a. Typical dimensions of each sample were kept at around 3.5 × 2.5 × 2.5 mm. The material behavior was tested through stress relaxation tests by applying a compressive strain and measuring the compressive stress development in a sample over time using an in-house built setup; see Figure 2a. The setup’s level of accuracy is discussed in the Text S2. Each sample was made to undergo a rapid compressive deformation of 20% of its initial length using a high-speed motorized stage (1 mm/s), after which this deformation was kept constant for 350 s, and a time-series data of the stress generated in the sample was logged-on to a PC from an integrated force sensor; see Figure 2a. For one of the materials (FF-AMS-152 with 40% magnetic content), we also carried out quasi-static compression experiments, and the result was consistent with that of the stress relaxation tests, as shown in Figure S4.

**Figure 2:**
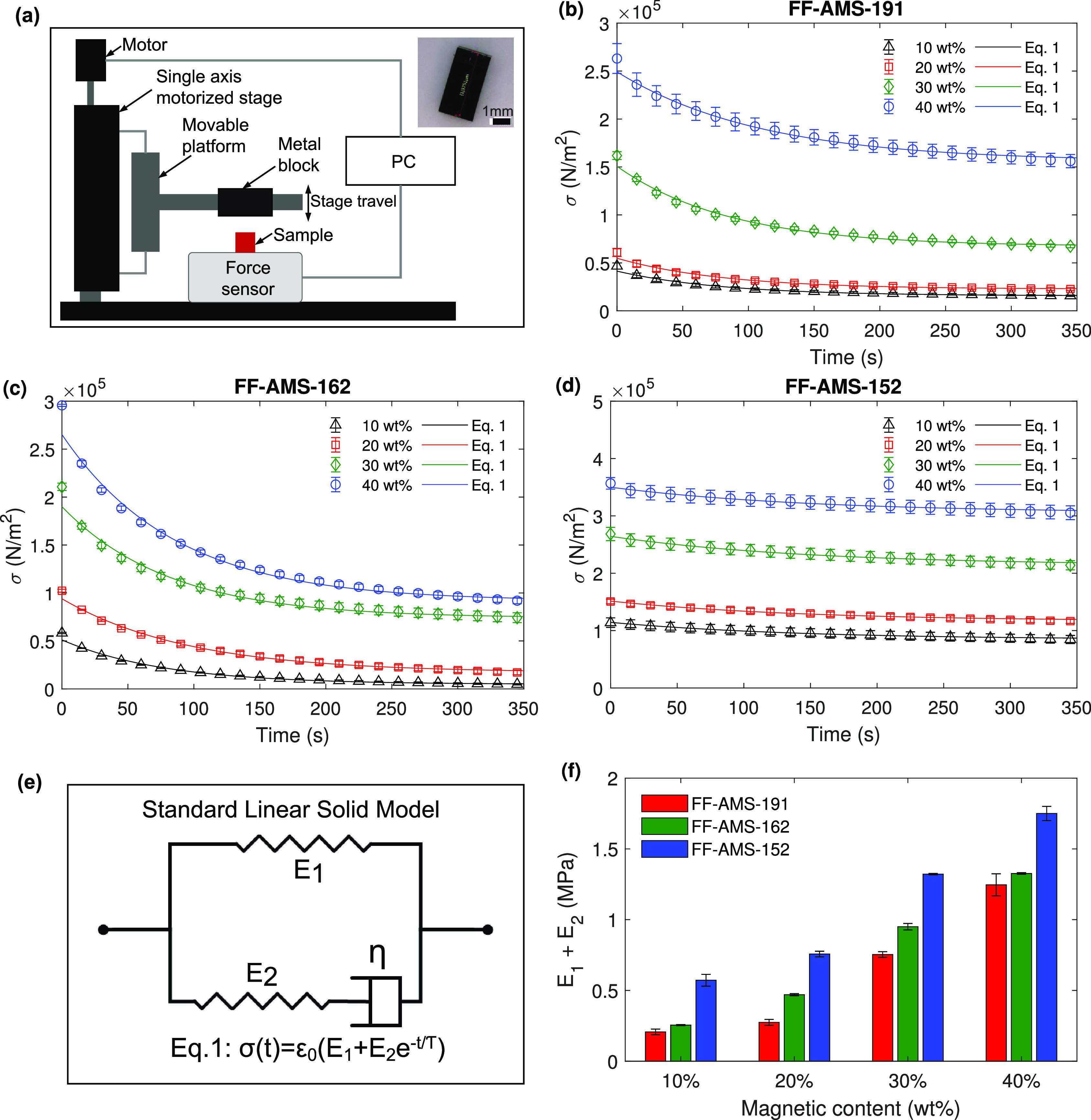
Material visoelastic properties: (a) Schematics of a custom-built setup used for performing the compression test on the synthesized nanomagnetic elastomers. A motorized stage moves a metal block mounted on a post connected to the stage platform, to compress the sample. The block is brought in contact with the sample first, which is detected by the fluctuation in the force sensor reading, and then compressed by 20% of its length by setting a travel length in the motorized-stage operating program. This compression is maintained at 20% for 350 s during which the change in force is recorded. The original length of each sample is measured by a contactless method using a microscope with integrated length measuring features (from Keyence). The inset image shows a cut sample. (b) Stress relaxation in the nanomagnetic elastomer FF-AMS-191 with different magnetic particle concentrations shows a quick decay first and then tends to stabilize over a time period of a few minutes. The behavior is represented by the standard linear solid model of the viscoelastic materials, and the model parameters fitted to the measured data are summarized in Table 1. The error bars in the figure are from the average of three measurement points. (c,d) Stress relaxation of the polymers FF-AMS-162 and FF-AMS-152 shows a different decay and stabilization profile as captured by the fitted model parameters shown in Table 1. (e) The data from the force sensor (with a sampling time of 1 s) were fitted with the viscoelastic model given by eq 1, the standard linear solid model, as schematically represented here. The coefficient of determination (*R*^2^) for all of the fits shown in parts b-d is above 0.97. (f) The instantaneous Young’s modulus of the materials, which is the sum of the two stiffnesses of the springs in the viscoelastic model, shows an increasing trend with an increase in magnetic content (wt %) as well as with an increase in the molecular weight of the polymers for the same magnetic content.

Considering the nanomagnetic elastomer with the lowest molecular weight FF-AMS-191 first, see Figure 2b, four samples with a magnetic content of 10%, 20%, 30%, and 40% by weight were tested. Compressing and holding the samples at a constant strain (ε_0_) of 0.2, all four samples show a relaxation/decrease in the generated stresses (σ(*t*)), therefore characterizing them as viscoelastic materials. The stress relaxation rate (−dσ(*t*)/d*t*) decreases with time and the stress tends to stabilize at a nonzero value as shown in Figure 2b. This measured behavior was further quantified by applying the standard linear solid model, a viscoelastic constitutive model, which is analogous to a spring-dashpot system where a spring and a dashpot are connected in series and a second spring is connected in parallel to them, see Figure 2e.^38^ The stress variation over time (σ(*t*)) in a material loaded at constant strain ε_0_ as per the model is therefore given by
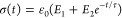
1where the term τ = η/*E*_2_ is the relaxation time of the material. An analysis of the equation shows that the sum of the moduli *E*_1_ + *E*_2_ represents the instantaneous elastic response at short time scales, i.e., much shorter than τ, which is relevant for fast and dynamic loading of the material. The long-term elastic response is represented by the modulus *E*_1_, which is relevant for slow, static, and long-term loading times larger than τ. When loaded over time, the elastic energy of the spring element *E*_2_ is released to restore the dashpot element to its original state; hence, both *E*_2_ and η determine the relaxation time scale τ. By fitting the model of eq 1 to the stress relaxation data obtained from the experiments, as shown in Figure 2b–d, all the model parameters were evaluated; see Table 1. Similar relaxation times (τ) for all four concentrations of each nanomagnetic elastomer in Table 1 suggest that the increase in viscosity (η) is proportional to the change in elasticity (*E*_2_). On the other hand, the stress relaxation rate, obtained by differentiating eq 1 with respect to time, is inversely proportional to the time constant (τ). Increasing *E*_2_ leads to an even faster increase in the stress relaxation rate, which scales with the square of *E*_2_; this is evident from Figure 2b–d and Table 1. The relaxation time (τ) obtained from the analysis ranges from 70 to 150 s for all the nanomagnetic elastomers which is much higher than the typical frequency at which the materials are actuated in micro- and nanoapplications similar to the demonstrations discussed below. The elastic behavior represented by the two combined stiffnesses (*E*_1_ + *E*_2_), representing the instantaneous Young’s modulus shown in Figure 2f, shows an increase with particle concentration which is due the reduced amount of free base polymer molecular chains existing in the material. Similar to the elastomer FF-AMS-191, the other two prepared nanomagnetic elastomers FF-AMS-162 and FF-AMS-152 with higher molecular weights also show viscoelastic behavior, see Figure 2c, d. The elastic behavior (*E*_1_ + *E*_2_) of both the elastomers again shows a gradual increase with the increase in the magnetic content from 10% to 40%, see Figure 2f. For a particular concentration, the elastic modulus *E*_1_ + *E*_2_ shows an increasing trend with respect to the molecular weight of the base polymer used, as shown in Figure 2f. The elastic modulus of all nanomagnetic elastomers with 40 wt % magnetic content was also evaluated under the influence of an applied magnetic field of around 200 mT during compression, see Figure S5a, b. The presence of the magnetic field resulted in an increase of the modulus by 6% to 16%.

The magnetization behavior of the nanomagnetic elastomers was characterized by using SQUID (superconducting quantum interference device) measurements. The magnitude of force that can be generated by a magnetic material is directly proportional to the volume of the magnetic content present in it. Figure 3a shows the measured magnetization curve of the polymer type FF-AMS-191 at 40 wt % magnetic particles; the inset of the plot shows the low hysteresis, indicating the superparamagnetic nature of the nanomagnetic particles in the elastomer. Measurements done on the nanomagnetic elastomers with a magnetic content of 10%, 20%, 30%, and 40 wt % again show a typical superparamagnetic behavior with linearly increasing magnetization values with increasing particle concentrations as shown for the lowest molecular weight elastomer FF-AMS-191 in Figure 3b. Similar results were obtained for the other two elastomers with higher molecular weights (FF-AMS-162 and FF-AMS-152) as shown in the supplementary Figure S6 and S7. This linearity is more evident in Figure 3c, which shows the mass magnetization at 100 mT for all three polymer types. Results at 5T are shown in Figure S8. Consistent magnetization profiles and saturation values of all three elastomers prepared with the same magnetic content indicate that our material synthesis process enables precise particle concentration tuning.

**Figure 3:**
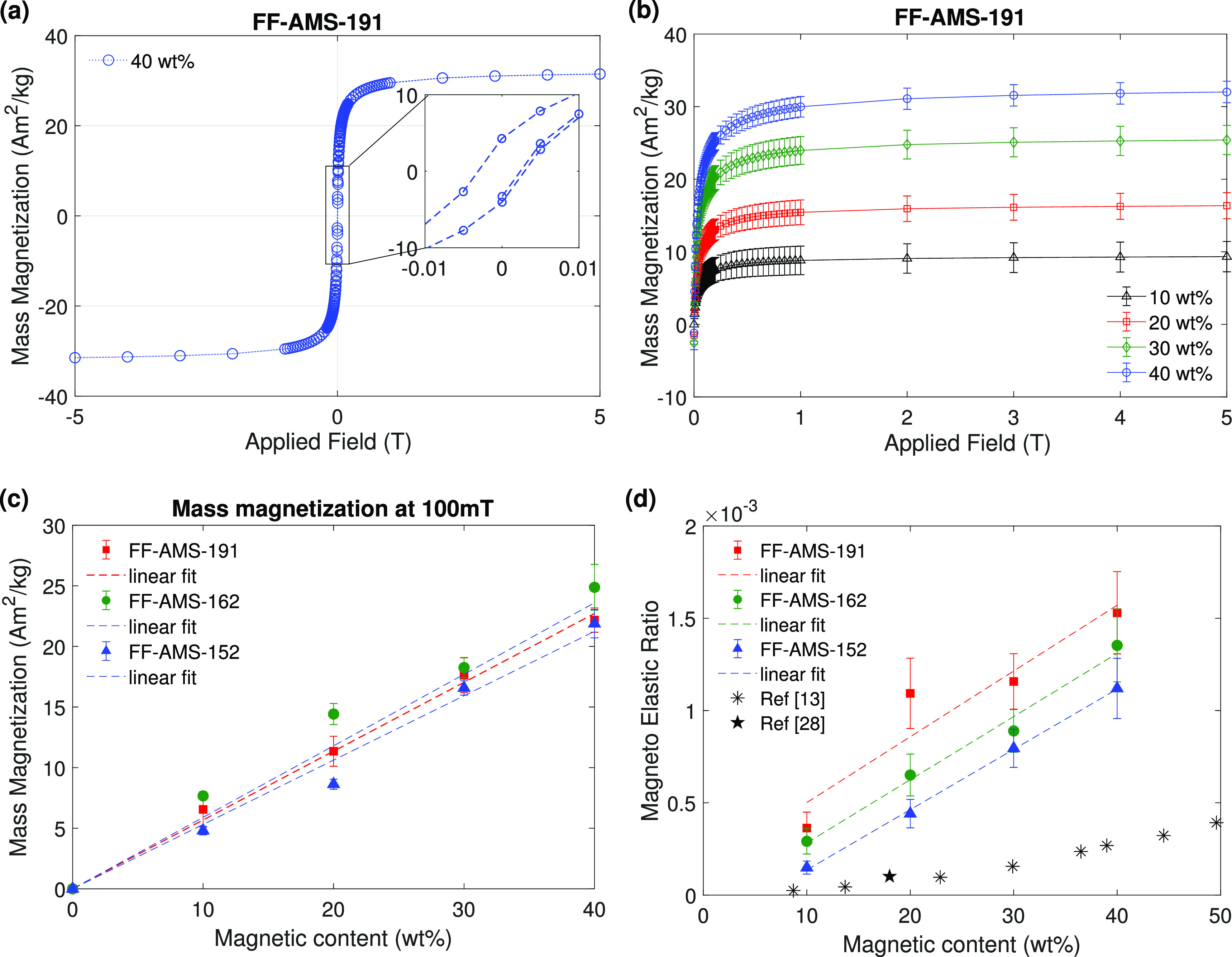
Material magnetization: (a) Magnetization curve of the nanomagnetic elastomer FF-AMS-191 at 40 wt %. The inset plot shows a very small hysteresis present in the material. (b) The mass magnetization curves of the FF-AMS-191 nanomagnetic elastomer for different magentic particle concentrations, showing the increase in the magnetization saturation value with the increase in the particle concentration. The curves for FF-AMS-162 and FF-AMS-152 are shown in Figures S6 and S7. (c) Mass magnetization values at an applied field of 100 mT, which is the typical value at which the magnetic materials are actuated in micro- and nanoapplications, increase linearly with the magnetic particle concentration for all three polymer types. The mass magnetization at 5 T is shown in Figure S8. (d) A linear increase with magnetic content in the magnetoelastic ratio (MER) values of the prepared nanomagnetic elastomers represents a balanced increase in stiffness and magnetization with the increase in magnetic concentration. All the MER values are calculated with the mass magnetization (*M*) at 300 mT. All previously reported nanomagnetic elastomers with uniformly distributed particles at nanoscales have their MER values lower than the ones we report here, except for one material^14^ which has a MER equivalent to FF-AMS-162 with 40 wt % magnetic particle concentration. Existing materials show a rapid increase in their stiffness with higher magnetic concentration, therefore reducing their MER; as an example, the asterisk (*) symbol shows data (calculated for the no-magnetic-field modulus) from ref (13). Another process that was previously reported for preparing a nanomagnetic material, represented by the star symbol, enabled the preparation of only low magnetic concentration elastomer, which gave it a low MER value.^28,29^

Increase in the magnetization, due to the increased magnetic content, goes hand in hand with the increase in the material Young’s modulus as shown above. The extent to which an applied magnetic field can overcome the elastic force in a cantilever-like structure to actuate or deform it, is determined by the magnetoelastic ratio given by^13^
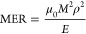
2where μ_0_ is the permeability of the free space, *M* is the mass magnetization of the material, ρ is the material density and *E* is the material elastic modulus (*E*_1_ + *E*_2_). The MER values of all the three nanomagnetic elastomers show a linearly increasing trend with particle concentration, with the rising graphs being parallel to each other; see Figure 3d. The lowest molecular weight nanomagnetic elastomer FF-AMS-191 shows the highest MER value at the maximum magnetic content of 40 wt %, whereas the lowest MER value is shown by the highest molecular weight nanomagnetic elastomer FF-AMS-152 at the lowest magnetic particle concentration of 10 wt %, see Figure 3c. The increase in MER with magnetic particle concentration is caused by the fact that the elastic modulus increases less strongly than the magnetization, and this is why the developed synthesis process can successfully produce a high concentration and highly responsive nanomagnetic elastomers. The MER values of other nanomagnetic elastomers reported in the literature so far are also included in Figure 3d for comparison. Only two earlier studies are known to us that report materials with sufficient nanoscale homogeneity in magnetic particle distribution to allow for fabricating actuatable submicron structures,^13,28,29^ Clearly, the magnetoelastic ratio of our nanomagnetic materials is substantially higher. Additionally, Figure S5c presents a comparison of the magnetoelastic ratio (MER) of all the materials with 40% magnetic content, obtained with and without the application of a magnetic field of approximately 200 mT. The presence of the magnetic field reduces the MER by 6 to 16%.

To experimentally verify the responsiveness of all the materials and their ability to produce highly compliant systems, the bending response of artificial micro- and nanocilia was tested next. Artificial micro- and nanocilia have diameters, their smallest feature, that are larger and smaller than 1 μm respectively, and they work as micro/nano actuators or sensors mimicking biological cilia.^5^ Employing a template based artificial cilia fabrication process,^14^ we fabricated magnetic artificial cilia with a nominal radius of 1.5 μm and length 47 μm (aspect ratio of 15.6) using our nanomagnetic materials. The artificial cilia were actuated using the setup schematically depicted in Figure 4a. Two opposing magnets generate a uniform magnetic field, and the cilia were placed in the center; to achieve maximum bending of the cilia, the applied magnetic field was chosen close to the saturation magnetization value, i.e., 200 mT. Due to the magnetic torque acting on the cilia they will tend to align with the magnetic field, hence they bend. Rotating the magnets clockwise as indicated in Figure 4a, the cilia bend to the right until the magnetic torque is not sufficient anymore to counter the elastic torque caused by the elastic energy increase due to bending, and the cilia whip back to their upright shape, see Movie S1. At the critical angle at which this happens, the bending is strongest; ultimately, the cilia tip may touch the surface, and the cilia reach the maximum bending that is achievable. The critical angle and corresponding bending are measures of the responsiveness and compliance of the cilia. The expectation is that the critical angle is larger and the bending is stronger for cilia made of materials with higher MER. This is confirmed by the results depicted in Figure 4b–e, showing the bending of cilia close to the critical angle for two nanomagnetic materials (FF-AMS-152 and FF-AMS-162), both at two magnetic particle concentrations (30% and 40%). The cilium made from FF-AMS-152 at a particle concentration of 30%, with a relatively low MER of the materials used here according to Figure 3d, shows relatively weak bending and a small critical angle. The cilium made from FF-AMS-162 at particle concentration 40%, with a higher MER, shows stronger bending and the highest possible critical angle—the cilia tip touches the surface during ultimate bending. The latter illustrates that indeed our materials can give extreme actuation due to their high compliance and high magnetization.

**Figure 4:**
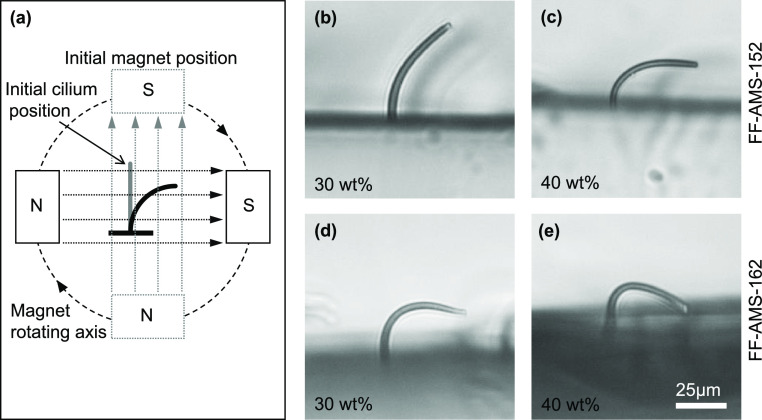
Magnetic artificial cilia: (a) Schematics of the cilia actuation setup where two permanent magnets create a nearly uniform and parallel field around the cilia to be actuated. The magnets are mounted on a shaft that is rotated about the central axis by a custom-built belt and pulley mechanism driven by a DC motor. The magnetic field is initially aligned parallel to the cilia length, and rotating the magnets clockwise rotates the magnetic field around the cilia which experience a magnetic torque and undergo bending to the right, see Movie S1. (b and c) Maximum bending achieved of cilia with radius 1.5 μm and length 47 μm, right before they whip back, made from the highest molecular weight elastomer FF-AMS-152 with 30 and 40 wt % of the magnetic nanoparticles. Increased magnetic content increases the maximum bending angle in agreement with their increasing MER values. (d) Cilium made from the second highest molecular weight elastomer FF-AMS-162 with 30 wt % of the magnetic content shows higher bending than the cilium with the same magnetic content made from the highest molecular weight elastomer FF-AMS-152. This is again in line with the MER values of the respective nanomagnetic elastomers. (e) Maximum possible bending, where the cilium tip touches the base surface, is already achieved for a cilium made from FF-AMS-162 with 40 wt % of the magnetic particle content. Since the elastomer with the lowest molecular weight FF-AMS-191 has an even higher MER value, its compliance/responsiveness is even higher and it therefore shows the same maximally possible bending behavior, i.e. the cilium tip touches the base surface like in (e).

We exploited this extreme compliance and high magnetization to achieve bending and actuation within a liquid environment in a 360° rotary motion of densely packed microcilia made from FF-AMS-191 with 40% magnetic particle concentration and with nominal radius 1 μm and length 23 μm. Indeed, Movie S2 shows that we obtain a fast and substantial response when applying a horizontally rotating magnetic field of ∼150 mT. Actuation of such densely packed cilia with an average gap of just 6 μm has not been achieved with any other nanomagnetic elastomer reported so far. Closely spaced magnetic structures within liquid environments interact magnetically as well as hydrodynamically, which hampers their responsiveness. A very high MER of a material is required to produce actuation in such densely packed magnetic structures. With large gaps between similar cilia structures made from a nanomagnetic elastomer with lower MER, the induced magnetic force is strong enough to make cilia undergo maximum bending at an angle of ∼90° and actuation at high frequencies as shown in Movie S3 for cilia made from FF-AMS-162 with 40% magnetic particle concentration.

Toward confirming the nanoscale applicability of the developed materials, we fabricated artificial cilia with a radius of only 350 nm and length of 9 μm (aspect ratio of 12.8); the material used was FF-AMS-162 with 40% magnetic particle concentration. By applying a rotating magnetic field of ∼150 mT these nanocilia indeed show extreme deflection and rotation with the field for a range of frequencies, see Movie S4. Successful actuation of nanostructures made from the prepared materials confirms the nanoscale homogeneity of the dispersed particles in the elastomer. The observed large bending and high frequency actuation of the fabricated micro- and nanostructures firmly validate the strong response and large compliance of the prepared nanomagnetic elastomers.

To conclude, we have developed a material synthesis process that enables the preparation of nanomagnetic elastomers with increased magnetic nanoparticle concentration and corresponding magnetization while maintaining a lower elastic modulus compared to previously obtained materials. The material preparation was accomplished by bonding the magnetic nanoparticles with polymers of different molecular weights/chain-lengths by creating the right pH conditions using an ammonium hydroxide solution and adding the right quantities of the polymers to a particle containing solution. The bonding process has an efficiency of more than 90% and the resulting magnetic elastomers show a uniform dispersion of the particles all the way down to nanoscales. Compression relaxation testing revealed the viscoelastic nature of the nanomagnetic elastomers with typical relaxation times of around 100 s. The instantaneous elastic Young’s modulus showed an increase with magnetic particle concentration as well as with the molecular weight of the base polymers. We characterized the magnetization of the materials through SQUID measurements and found a linear increase with the magnetic particle concentration. From the measured elastic and magnetic properties, we determined the magnetoelastic ratio (MER). The values obtained were found to be substantially higher than those reported earlier for previously developed nanomagnetic elastomers, underlining that our materials have uniquely strong magnetic properties combined with high compliance. We further demonstrated that our nanomagnetic elastomer with a higher MER could be used to fabricate dense carpets of microscopic artificial cilia that could be actuated strongly within a liquid environment by a rotating magnetic field. Such motions at the micro- and nanoscale and at such dense configurations are observed commonly in nature, but artificially mimicking them had not been realized before due to the complexities involved in achieving the right materials. The process we have reported here to achieve such functional materials may well be only the beginning of a further development toward a wide range of applications for sensing and actuation in micro- and nanoscale systems.

## Supporting Information Available

Detailed explanation of the material synthesis process specifying the details of the materials used, as well as extended information about the setup employed; SEM and AFM images of the particle distribution in the polymer matrix; figure that presents a comparison between quasi-static and instantaneous compression tests; additionally, it provides data on the elastic modulus and MER measured while applying a magnetic field; figures with additional information about the magnetization of the materials; and a detailed description of the micro- and nanocilia structures seen in the supplementary movies (PDF)
Movie S1: Side view of a magnetic artificial microcilia showing bending and elastic recovery as the magnetic field continues to rotate beyond the bending limit of the cilia (MP4)
Movie S2: The possibility to actuate dense carpet of magnetic artificial microcilia using the material with highest magneto elastic ratio (MP4)
Movie S3: Sparsely spaced magnetic artificial microcilia showing extreme deflection while they are actuated at different frequencies (MP4)
Movie S4: Actuated magnetic artificial nanocilia with radius 350 nm demonstrating the nanoscale homogeneity of the materials prepared in this work (MP4)
