# Supplementary material for: Nanomagnetic Elastomers for Realizing Highly Responsive Micro- and Nanosystems
Source: Nano Lett. 2023 Jul 19;23(20):9203–11. doi: 10.1021/acs.nanolett.3c00819 (PMC10603798; doi:10.1021/acs.nanolett.3c00819)
Supplement: Supplementary file 1 — nl3c00819_si_001.pdf [file nl3c00819_si_001.pdf]

Supporting Information

Nanomagnetic elastomers for realizing highly responsive micro- and nanosystems †

Bhavana B. Venkataramanachar,‡,¶ Jianing Li,§ Tanveer ul Islam,‡,¶ Ye Wang,‡,¶ and Jaap M. J. den Toonder*,‡,¶

‡*Microsystems Section, Mechanical Engineering, Eindhoven University of Technology, 5612 AZ, The Netherlands*

¶*Institute for Complex Molecular Systems, Eindhoven University of Technology, 5612 AZ, The Netherlands*

§*Department of Applied Physics and Science Education, Eindhoven University of Technology, 5612 AZ, The Netherlands*

E-mail: J.M.J.d.Toonder@tue.nl

This PDF file includes:

Supplementary Text S1 and S2

Figures S1 to S8

Captions for Movies S1 to S4

References 1 to 5

Other Supplementary Materials for this manuscript include the following:

Movies S1 to S4

†A footnote for the title

Text S1: Material synthesis process

Materials required for the nanomagnetic elastomer preparation are: reagent-grade 97% ferric chloride ($FeCl_3$), reagent Plus 98% ferrous chloride tetrahydrate ($FeCl_2H_8O_4$), ammonium hydroxide solution (NH_4OH) containing 28% NH_3 in H_2O, chloroform with 0.5 to 1% ethanol as stabilizer, and 98% dicumyl peroxide from Merck−Sigma Aldrich. Polymer AMS-191, AMS-162 and AMS-152 containing $9-11\,\%$, $6-7\,\%$ and $4-5\,\%$ of aminopropylmethylsiloxane with dimethylsiloxane, respectively, from Gelest, Inc. The ferric and ferrous chloride salts are stored in a dry glovebox (Inert Laboratory 2 GB) to prevent them from moisture.

The synthesis of nanomagnetic elastomers consists of the steps shown in Fig. S1. First, is the preparation of magnetite nano particles from iron salt in an aqueous solution[1–3] and second, coating the particles by a polymer followed by their separation from the solution and the uncoated particles and finally tuning of their concentration to yield a nanomagnetic material. The entire process is carried out in a custom-built setup inside a fumehood. The step-by-step procedure is detailed below:

- ❏ Ferric and ferrous chloride solutions are prepared by adding 3.64 gm of former and 2.23 gm of the latter salt to 60 ml of DI water separately in two glass beaker. The salts are taken from the glovebox in glass-vials and then directly added to the water beaker placed on a weighing scale.

- ❏ The salts are stirred using a glass rod for around $5-10$ minutes and mixed together in a 200 ml capacity glass bottle/ vessel (from VWR). The mixed solution has two salts in a molar concentration ratio of 2:1 (ferric to ferrous chloride).

- ❏ The reaction vessel containing the solution is closed with a $GL-45$ cap (from VWR) modified to hold four transfer tubes (from Tygon) and a DC motor (DCX 19 S from Maxon) at its center as shown in Fig. S2. Three of the four tubes have an external diameter of 3 mm and the fourth one has a diameter of 5 mm. The three smaller tubes

extend into the vessel till around 1 cm above the final solution level while as the fourth tube stays very close to the inner surface of the cap, extending only few millimeters inside. To ensure leak-tight tubing in the cap, the holes are made 100 μm smaller than the tube diameters. The motor at the center is secured to the cap using screws with a sealing tape placed between the motor and the cap. The top side of the motor is closed off with a PMMA cap and sealed using glue to prevent leakage through the motor. The sealing tape is prepared using a laser cutter with the right size and location of the holes for the motor shaft and the screws to pass through. The PMMA cap is made using a micro-milling machine. The motor shaft is connected to a glass stirrer through a push-fit assembly using a teflon-tape layer in between. The tube opposite the biggest diameter tube is connected to a nitrogen supply and one of the other two tubes is connected to a burette. All the tubes are fitted with a luer-lock or valve at the end.

- ❏ After the setup, the vessel is flushed with nitrogen for 30 minutes to reduce the oxygen concentration to a minimum level. During nitrogen flushing, only the biggest diameter tube is kept open as it removes the air by collecting it at the topmost level, being close to the inner side of the GL − 45 cap. The other two tubes are opened and closed for few seconds after every 10 minutes to ensure no oxygen is trapped in the tubes.

- ❏ After 30 minutes of flushing, the burette is filled with ammonium hydroxide solution and the stirrer is turned on. The stirrer speed is set between 300 − 400 rpm.

- ❏ Stirring for a few minutes, the solution is titrated with the ammonium hydroxide solution to precipitate the nanomagnetic particles. The amount of NH_4OH added to the solution depends on the type of polymer to be used for coating the particles, see Fig. S1. The quantity of nanomagnetic particles produced is known by balancing the particle synthesis reaction.[4]

- ❏ The titration is followed by stirring the solution for around 10 minutes and then a polymer is added using a glass syringe. The amount of polymer added in case of

AMS-152, AMS-162 and AMS-191 is 2 ml, $2-3$ ml and 6 ml respectively.

- All the tubes are closed and the nitrogen supply is stopped, isolating the environment inside of the reaction vessel from the outside oxygen-rich environment. The solution is left to stir for the next $14-16$ hrs to allow particle-polymer bonding.

- With sufficient time given for bonding, the stirring is stopped and the cap is removed. The magnetic nanoparticles along with the polymer, forming a thick black phase, are settled using a permanent magnet and the clear solution left above is decanted. Particles that tend to run away in the end with the decanting solution are held back by letting a small volume of the solution to stay back.

- To separate the bonded/ unbonded particles from the extra polymer and the small amounts of the by-products, around $200\,ml$ methanol is added to the particle-polymer complex and the content is stirred using a glass rod for $5-10$minutes. The particles are again settled on a permanent magnet and the clear content left above is decanted. This step is followed two more times but with a smaller quantity of methanol $\sim 150\,ml$. In the third step of methanol addition, the stirring rod is rinsed/ washed with methanol and the solution is stirred without the glass rod.

- Continuing without the glass rod, the methanol is decanting and around $150\,ml$ of DI water is added. Stirring turns the particle-polymer complex into lumps/ flakes floating in the added water. Use of stirring rod with water makes the lumps to stick to the rod which is difficult to rinse off later on. Stirring for a few minutes, the particles are settled again and the content left above is poured out. This step is also repeated three times.

- The water decanting is again followed by methanol addition and stirring for three more times, each time adding around $100\,ml$ of methanol. Methanol addition breaks down the lumps formed during water addition into finer fragments. The extensive process

of washing the particles with methanol and water removes all the extra polymer and other byproducts leaving behind all the coated as well as uncoated particles.

- To remove the uncoated particles, $30\,ml$ of chloroform is added and the vessel is closed with a new cap and the content is sonicated for half an hour. Sonication enables finer dispersion of the particles in the chloroform. The solution is now poured from the 200 ml vessel to smaller vials. The coated particles make a stable suspension with the chloroform whereas the uncoated particles are settled on a permanent magnet by letting them to settle for around $1\,hr$.

- The chloroform containing the coated particles are poured into another vial and the settled particles in the first vial are dried by letting the chloroform to evaporate in a fumehood.

- A small quantity of the particle-containing chloroform solution ($\sim 1\,ml$) is removed into a small glass vial (using a glass pipette) and its weight is measured.

- The chloroform is allowed to evaporate in a fumehood and the final quantity of coated particles left in the vial is compared with the initial quantity to calculate the weight of the coated particle content in the bulk of the chloroform solution.

- Knowing the quantity of particles produced from balancing the synthesis chemical reaction[4] and excluding the uncoated sediments, the quantity/ percentage of the particles and polymer in the chloroform solution is calculated. Typically, a maximum particle percentage of $40 - 50\,wt\%$ is obtained.

- To prepare a nanomagnetic elastomer from the ferrofluid, a required amount of ferrofluid is transferred to a vial and a measured amount of polymer is added, same as the coated polymer, depending on the concentration of the nanomagnetic elastomer to be prepared.

- Curing agent dicumyl peroxide equal to $10wt\%$ of the polymer present in the chloroform solution, including the coated quantity, is added and the solution is first stirred for a few minutes with a glass rod and then sonicated for half an hour.

- The solution is left for evaporation until all the chloroform evaporates leaving behind a uniform mixture of particles in the polymer $-$ a ferrofluid.

- The ferrofluid is either molded using polycarbonate track etched membranes (PCTE) to make cilia structures[5] or cured directly in the glass vial using a vacuum oven at $130\ ^0C$ for $8-9\ hrs$. The cured elastomer is removed from the vials and cut into rectangular sections to prepare samples of $3.5 \times 2.5\ \times 2.5$ mm for the compression test.

Text S2: Compression test setup accuracy

- The compression test setup consists of a motorized stage from Physik Instrumente model number M-405.DG, which is controlled by a DC motor controller C-863 from Mercury, and a force sensor from VWR with a model number LPW-303i. The minimum step size of the compressing stage is 0.001 mm, and the smallest force that can be measured is around 9.8 μN or 0.001g. These least count values of the components used are much smaller than the range of the applied strain and of the measured stress in this study, which gives the setup a high accuracy.

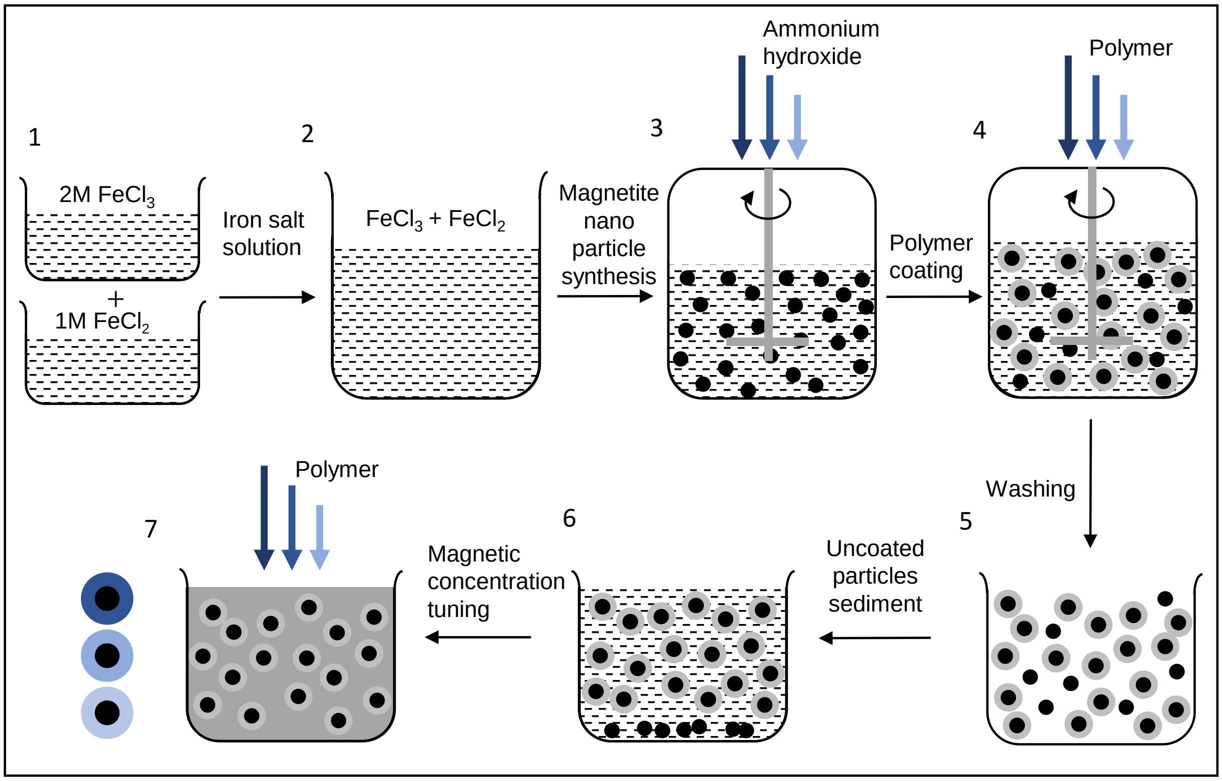

Figure S1: Schematic of the material synthesis process steps. Step 1: Ferric and ferrous chloride salts dissolved in deionized water. Step 2: both salt solutions are put into the reaction vessel. Step 3: precipitation of magnetite nanoparticles by titrating the salts with ammonium hydroxide in a sealed vessel. The blue arrows represent different amounts of ammonium hydroxide corresponding to different polymers used for particle coating. Step 4: coating of particles while they are being stirred continuously with the blue arrows showing the possibility to add varieties of AMS polymers (aminopropylmethylsiloxane with dimethylsiloxane), which vary in molecular weights. Step 5: removal of reaction byproducts and mainly the removal of excess polymer not used for coating through the use of solvents methanol and water. Step 6: separation of uncoated magnetic particles from coated particles suspended in chloroform. Step 7: tuning the concentration of the magnetic particles by weight with the same type of base polymer used to coat the particles.

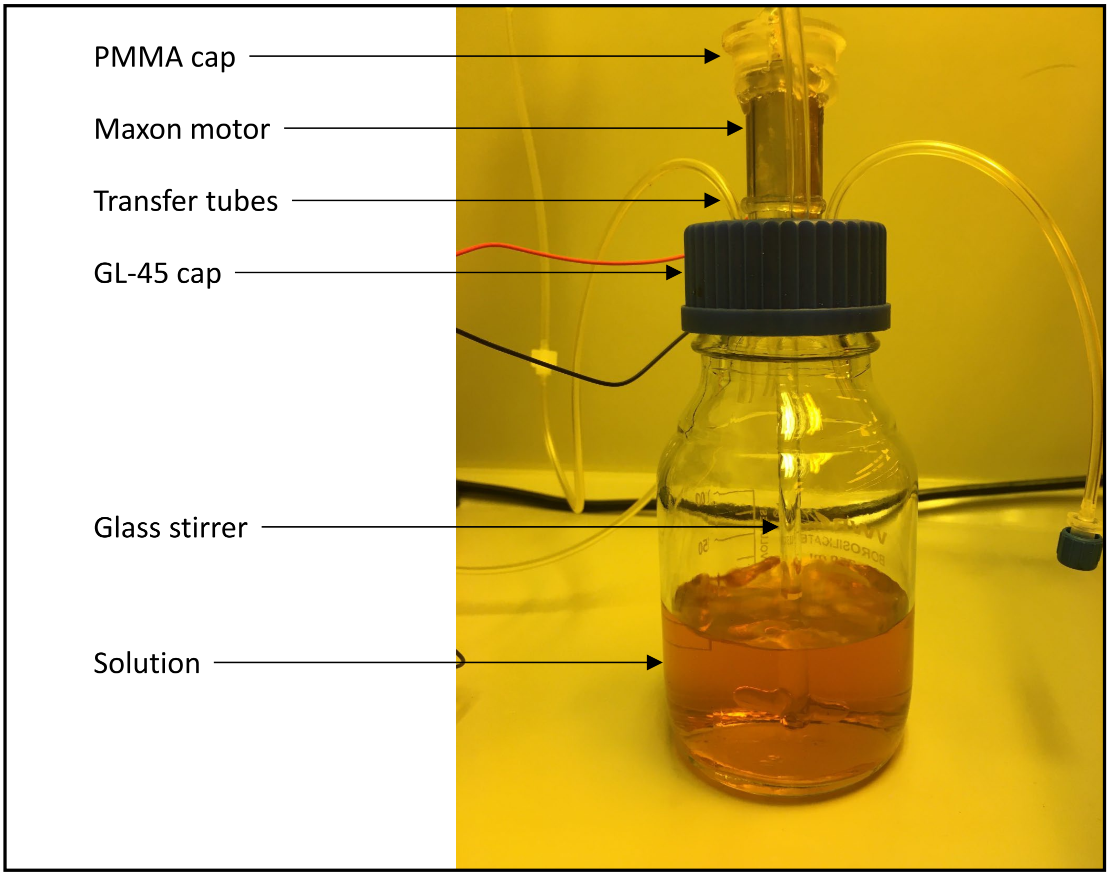

Figure S2: Custom-made setup for the material synthesis process: Among the different components labeled, the red color solution is a mixture of the two salts before the titrating step. The stirrer has one cross-link near the tip which is enough to stir the solution.

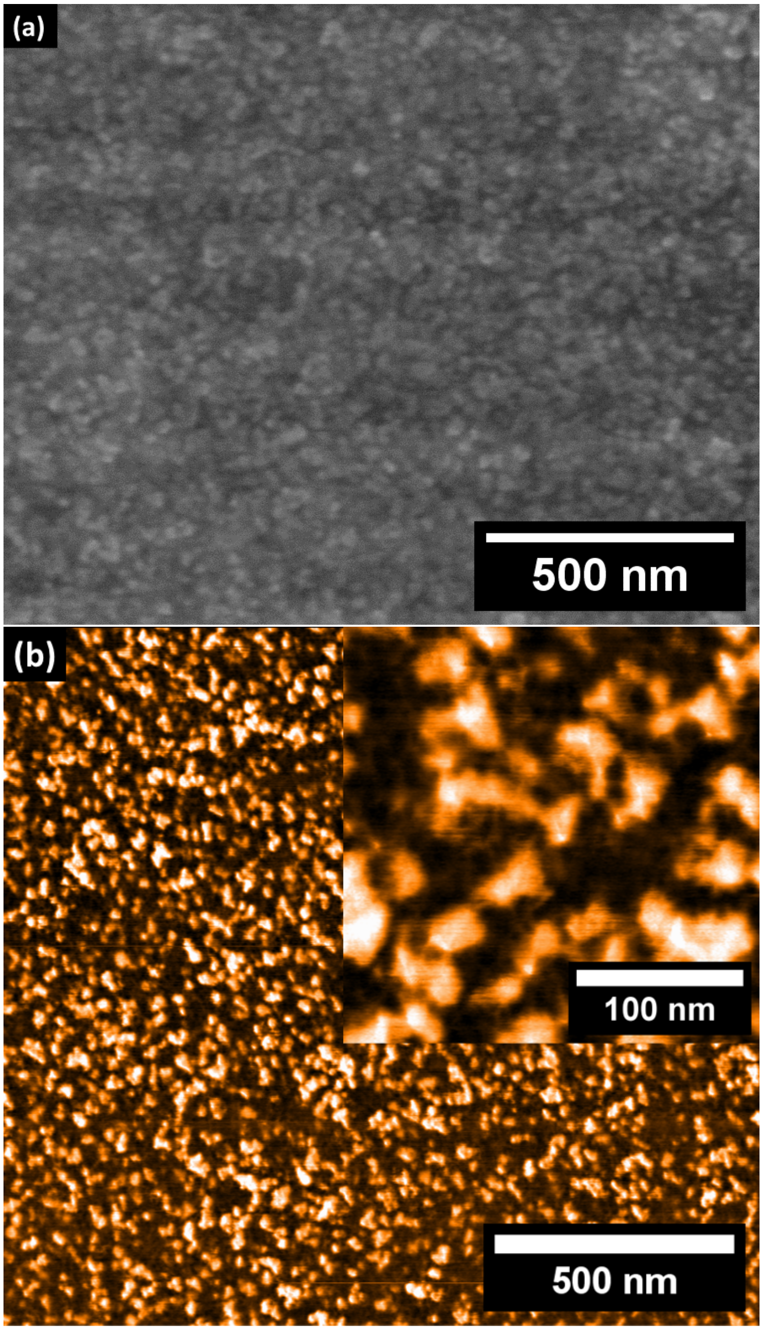

Figure S3: (a) A SEM (scanning electron microscopy) image of FF-AMS-191 40 wt% shows the uniform distribution of the coated magnetic particles in the polymer matrix. (b) An AFM (atomic force microscopy) image of the same magnetic polymer as in (a) obtained using phase contrast dynamic mode of the AFM. The particle size is around 20 nm.

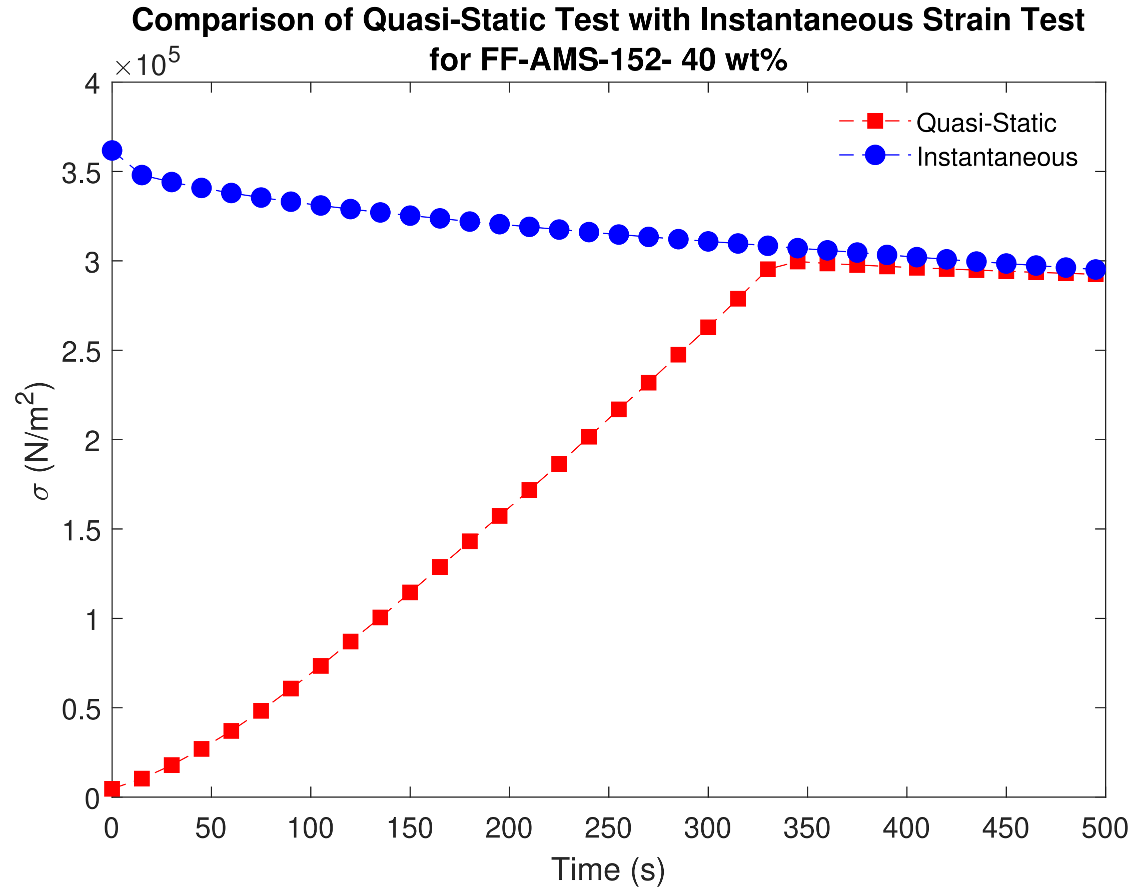

Figure S4: Comparison of a quasi-static compression test carried out using the setup shown in Fig. 2(a), for a sample from polymer FF-AMS-152 40 wt%, with the instantaneous strain test from Fig. 2(d). The quasi-static test is performed at a loading rate of 2μm/s up to a compressive strain of 20%, after which the strain is kept constant. At the loading rate of 2μm/s the sample compression of 20% is reached in a time interval which is larger than the time-constant (τ) of the same material obtained from the instantaneous strain test. The results from the two tests are consistent.

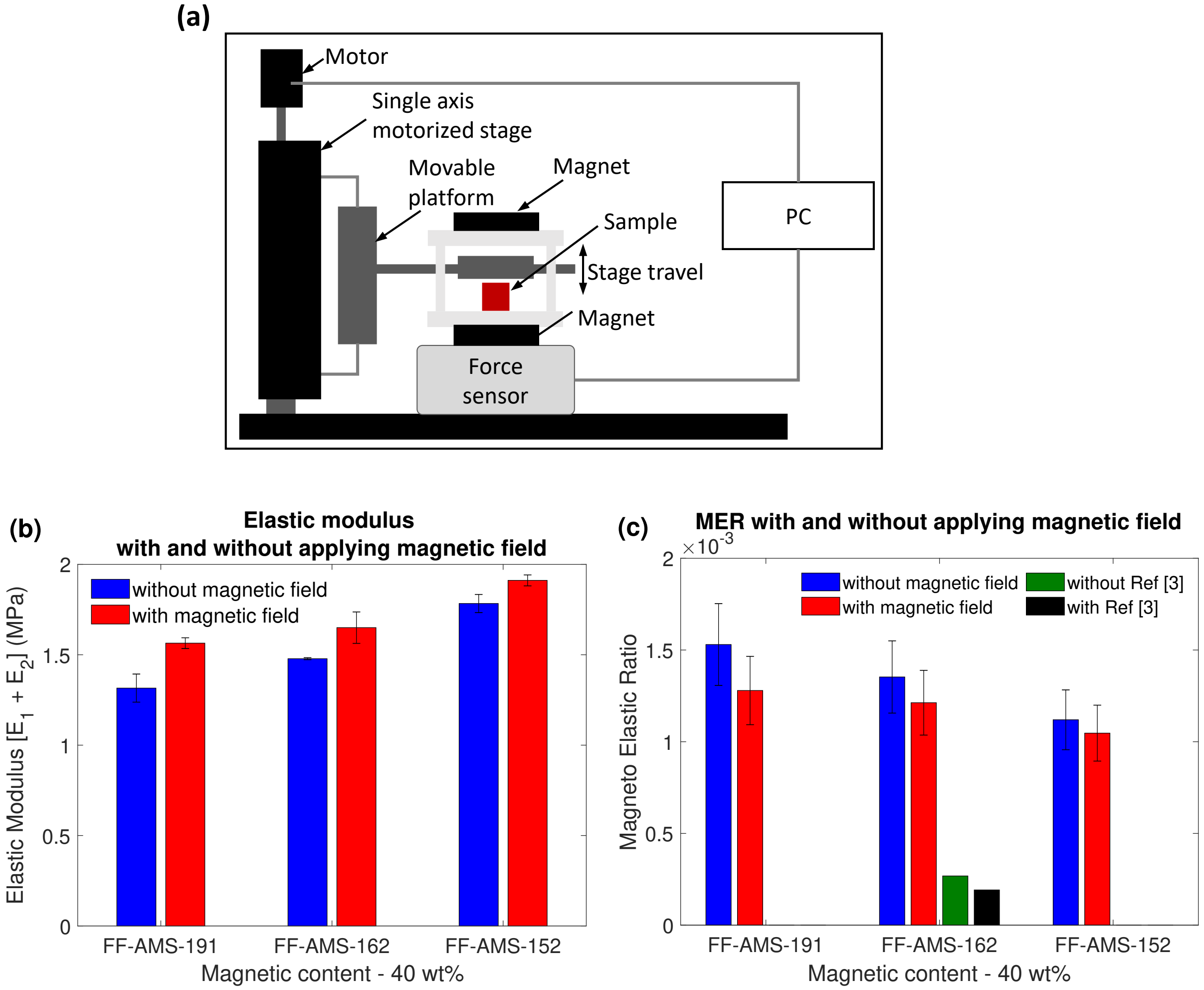

Figure S5: (a) Modified force measurement setup to study the elastic modulus of the nanomagnetic elastomers under the influence of a magnetic field. The sample is placed in a uniform magnetic field of around 200 mT in the loading direction, and is then compressed in the presence of this field according to the same procedure as used for the experiments without applied field. (b) The obtained elastic modulus for all the polymers at a magnetic loading of 40 wt%. There is an increase in the modulus by 6, 10 and 15% for FF-AMS-152, FF-AMS-162 and FF-AMS-191 respectively. (c) The corresponding differences in the MER due to the applied magnetic field, which decreases the MER by 6, 10, and 16% for FF-AMS-152, FF-AMS-162 and FF-AMS-191 respectively. Additionally, we compare the MER obtained from previous study of FF-AMS-162.[3]

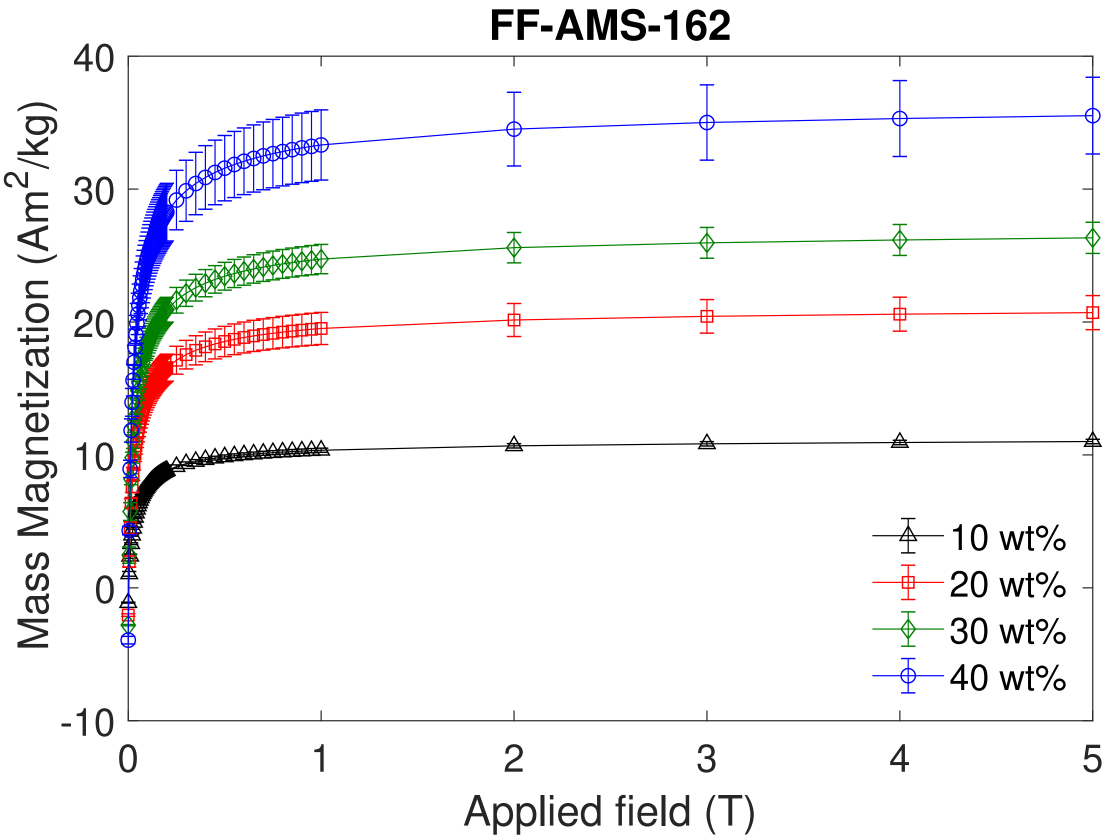

Figure S6: Magnetization curves of the nanomagnetic elastomer FF-AMS-162 with four different magnetic concentration from $10 - 40\ wt\%$. The curves are similar to the ones obtained for the polymer FF-AMS-191 at different concentrations.

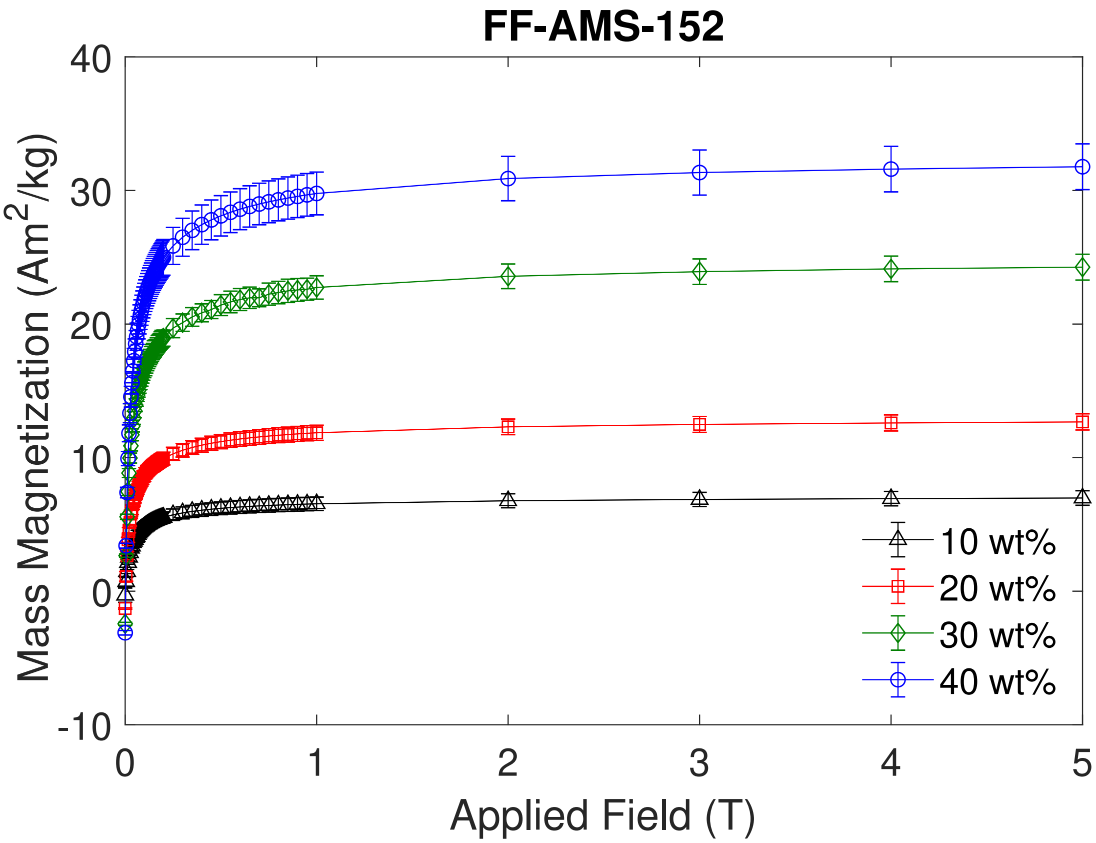

Figure S7: Similar to the magnetization curves obtained for the polymers FF-AMS-191 and FF-AMS-162 the magnetization curves obtained for the nanomagnetic elastomer FF-AMS-152 show an increasing saturation value for higher magnetic content.

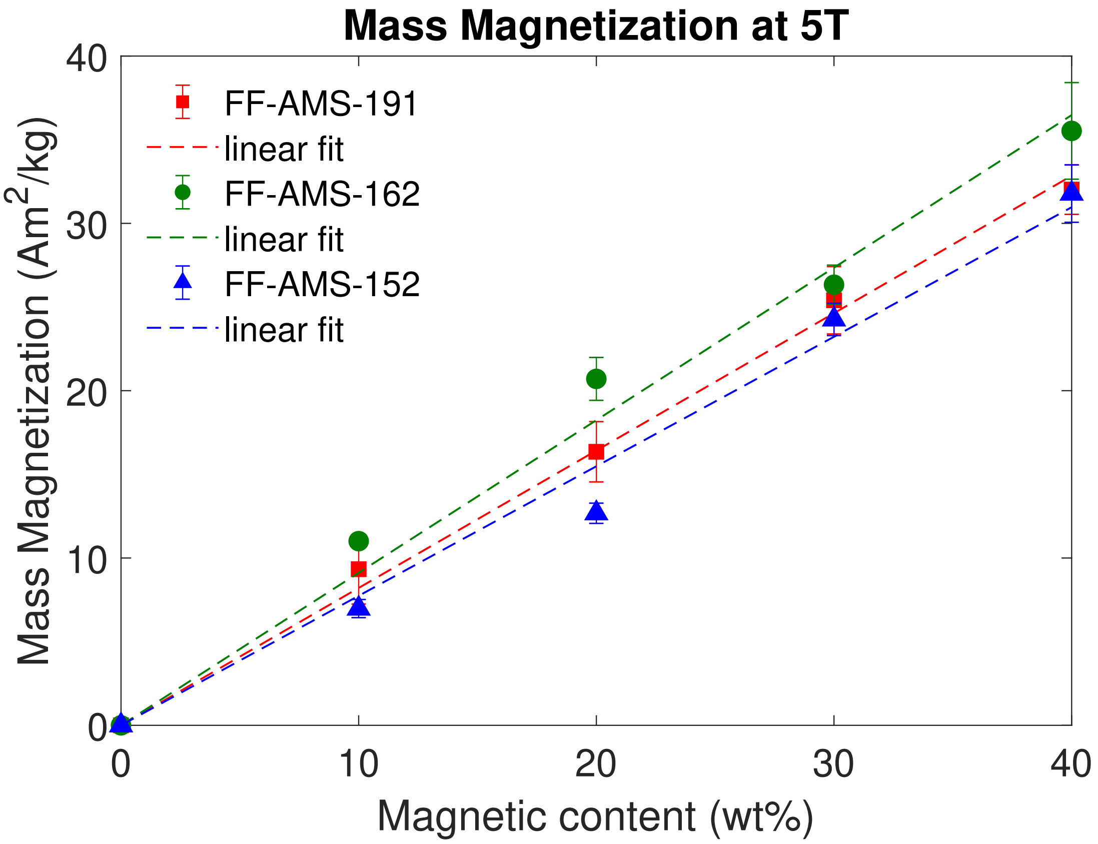

Figure S8: Similar to the linearly increasing mass magnetization values at 100 mT the saturation magnetization values also show an increasing trend for all the three nanomagnetic elastomer types with increasing magnetic concentration.

Movie S1: Side view of a magnetic artificial microcilia with radius 1.5 μm and length 47 μm showing bending and elastic recovery as the magnetic field of ~200 mT continues to rotate beyond the bending limit of the cilia. The elastomer used is FF-AMS-162 with 40 $wt\%$ magnetic particle concentration.

Movie S2: A dense carpet of magnetic artificial microcilia with radius 1 μm and length 23 μm with a distribution density of 3×10^6 cm^{-2} actuated with a horizontally rotating magentic field of ~150 mT at a frequency of $1 - 40$ Hz. Polymer used is FF-AMS-191 at 40 $wt\%$ magnetic concentration.

Movie S3: Sparsely spaced magnetic artificial microcilia with radius 1 μm and length 23 μm with a distribution density of 10^5 cm^{-2} showing extreme deflection and actuated at different frequencies of $1 - 40$ Hz, using a horizontally rotating field of ~150 mT. Polymer used is FF-AMS-162 at 40 $wt\%$ magnetic particle concentration.

Movie S4: Magnetic artificial nanocilia with radius 350 nm and length 9 μm with a distribution density of 10^5 cm^{-2} showing extreme deflection and actuated at different frequencies of $1 - 40$ Hz, using a horizontally rotating field of ~150 mT. Polymer used is FF-AMS-162 at 40 $wt\%$ magnetic particle concentration.

References

(1) Massart, R.; Dubois, E.; Cabuil, V.; Hasmonay, E. Preparation and properties of monodisperse magnetic fluids. *Journal of Magnetism and Magnetic Materials* **1995**, *149*, 1–5.

(2) Van Ewijk, G.; Vroege, G.; Philipse, A. Convenient preparation methods for magnetic colloids. *Journal of Magnetism and Magnetic Materials* **1999**, *201*, 31–33.

(3) Evans, B.; Shields, A.; Carroll, R. L.; Washburn, S.; Falvo, M.; Superfine, R. Magnetically actuated nanorod arrays as biomimetic cilia. *Nano letters* **2007**, *7*, 1428–1434.

(4) Widdrat, M. Formation and alteration of magnetite nanoparticles. Ph.D. thesis, Universität Potsdam, Mathematisch-Naturwissenschaftliche Fakultät, 2014.

(5) Islam, T. u.; Bellouard, Y.; den Toonder, J. M. Highly motile nanoscale magnetic artificial cilia. *Proceedings of the National Academy of Sciences* **2021**, *118*, e2104930118.
